# In-Situ Transformation of Li-ABW Zeolites Based on Li-Geopolymer

**DOI:** 10.3390/gels9050392

**Published:** 2023-05-09

**Authors:** Huaiyuan Dou, Quan Ye, Yan He, Xuemin Cui

**Affiliations:** Guangxi Key Lab of Petrochemical Resource Processing and Process Intensification Technology, School of Chemistry and Chemical Engineering, Guangxi University, Nanning 530004, China

**Keywords:** in-situ infrared spectroscopy, non-hydrothermal transportation, Li-geopolymer, Li-ABW

## Abstract

Lithium batteries, as energy storage devices, are playing an increasingly important role in human society. As a result of the low safety of the liquid electrolyte in batteries, more attention has been paid to solid electrolytes. Based on the application of lithium zeolite in a Li-air battery, a non-hydrothermal conversed lithium molecular sieve was prepared. In this paper, in-situ infrared spectroscopy, together with other methods, was used to characterize the transformation process of geopolymer-based zeolite. The results showed that Li/Al = 1.1 and 60 °C were the best transformation conditions for the Li-ABW zeolite. On this basis, the geopolymer was crystallized after 50 min of reaction. This study proves that the formation of geopolymer-based zeolite occurs earlier than the solidification of the geopolymer and shows that the geopolymer is a good precursor for zeolite conversion. At the same time, it comes to the conclusion that the formation of zeolite will have an impact on the geopolymer gel. This article provides a simple preparation process for lithium zeolite, explores the preparation process and mechanism, and provides a theoretical basis for future applications.

## 1. Introduction

Due to the problems of insufficient electrochemical and thermal stability, low ion selectivity, and poor safety in liquid electrolytes [1], solid electrolytes [2,3,4] that can replace liquid electrolytes have received increasing attention. Similarly, new energy vehicles have stricter safety requirements [5,6,7]. The International Energy Agency estimates that the global sales of electric vehicles will reach 245 million in 2030, on the basis of the current and expected future policies, with corresponding annual sales growth of approximately 41% and a stock value of USD 13 billion and USD 130 billion [8]. Thus, research on better solid electrolyte materials has become more significant.

Chi et al. [9] proposed a zeolite electrolyte solid-state Li-air battery. The LiX zeolite membrane is obtained by replacing the Li^+^ of the dense FAU molecular sieve membrane with Na^+^. However, from the perspective of the preparation process consumption, this requires a long time period for a hydrothermal reaction and multiple ion exchange to occur [10,11,12,13]. Therefore, it is more important to find a lithium zeolite with low energy cost and simple technology.

Metakaolin (MK)-based Geopolymer [14,15,16,17] has a suitable element ratio that can be crystallized and converted into zeolite in situ under certain conditions of temperature and pressure. Therefore, it is often used as the raw material of low-silicon zeolite. In addition, the advantages of this in-situ zeolite are that the geopolymer provides strength and stability, while the zeolite provides a high surface area, porosity, and adsorption [18].

The in-situ conversion of zeolite from geopolymers and its application have been widely studied [19,20,21]. For example, Ye et al. [22] used NaOH to activate MK foaming to prepare geopolymer microspheres and transform them into NaA molecular sieves in situ for CO_2_ hydrogenation catalyzed by supported Ni, Pt, and Pd. Guan et al. [23] used sodium silicate as the activator in the preparation of MK-based geopolymer; then, the faujasite zeolite was prepared through hydrothermal reaction in a 2 mol/L NaOH solution at 90 °C for 24 h. The compressive strength can reach 11.7 Mpa. Wei et al. [24] obtained the crystallinity of 62.42% cubic zeolite microspheres after curing the MK with 11 M sodium hydroxide at 85 °C for 72 h, and its adsorption capacity of Pb^2+^ was up to 308.3 mg/g. Such an approach manifests sustainability benefits as the synthesis of geopolymers is more energy- and time-efficient compared to conventional zeolite synthesis [18].

In terms of energy, compared with synthetic zeolite, the activation energy corresponding to the formation of low-silica zeolite was reduced by the geopolymer gel. In other words, the geopolymerization further ordered the arrangement of Si-O tetrahedron and Al-O tetrahedron and formed the microcrystals that would be seeds [25] in the in-situ transformation. Therefore, theoretically speaking, the crystallization of geopolymers should be milder. In addition, a NaA molecular sieve can be prepared using a MK-based geopolymer with a certain Na/Al ratio under non-hydrothermal conditions below 70 °C in the study of in-situ low-silicon zeolite [26]. However, crystallization of geopolymer usually requires hydrothermal conditions, which are no different from the conditions of artificial zeolite.

Lithium, the first element of the alkali metal group, has two hydroxides, both of which are strong bases that are rarely used in the activation of geopolymers because of their low solubility. In the relevant literature reports, lithium hydroxide (monohydrate) is generally used as the raw material of the in-situ lithium zeolite transformation precursor and as the solute of the hydrothermal solution. In general, the vast majority of lithium zeolite (almost zeolite ABW) prepared using the geopolymeric method [27,28,29,30,31] have been obtained from the hydrothermal reaction of solidified geopolymer blocks. However, the crystallization of a lithium-based geopolymer can be conducted at a low temperature and in a short amount of time, even without hydrothermal processes. In this work, a low-temperature and non-hydrothermal Li-ABW molecular sieve was successfully prepared. The optimum technology was explored by controlling different Li/Al ratios and curing (transformation) temperatures and times. The formation of Li-ABW was characterized using in-situ infrared spectroscopy. This work provides a simple preparation process for lithium zeolite, explores the preparation process and mechanism, and provides a theoretical basis for future applications.

## 2. Results and Discussion

### 2.1. XRD Analysis

As no hydrothermal reaction was required, only the effects of the Li/Al, temperature, and time on the crystallization of the lithium-based geopolymer have been investigated. The results were as follows.

**Figure 1:**
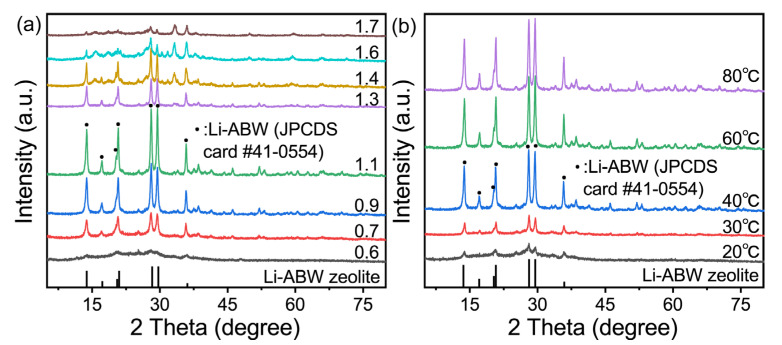
XRD patterns of effects of (**a**) Li/Al curing at 60 °C for 24 h and (**b**) temperature curing for 24 h when Li/Al = 1.1.

The curing condition was controlled at 60 °C for 24 h. Figure 1a shows the transformation of the geopolymer under different quantities of LiOH·H_2_O addition. What can be confirmed is that the characteristics diffraction peaks of all the crystalized samples at 2θ are 13.78°, 17.24°, 20.51°, 20.96°, 28.29°, and 29.65°, which are consistent with the standard card of Li-ABW zeolite (JPCDS card No. 41-0554). The crystallization rate shows a clear trend of first increasing and then decreasing with the increase in the Si/Al ratio. Generally speaking, when the amount of alkali added is small, the formation of geopolymer gel is insufficient, leading to a reduction in the raw materials of the subsequent molecular sieves, which causes a decrease in the output of zeolite. When Li/Al = 0.6, there were only weak diffraction peaks, and the sample was still an amorphous aluminosilicate gel, which can be proven. Similarly, if the alkalinity is too high, the zeolite microcrystals in the geopolymerization are dissolved by the high alkali slurry. However, when the activator is lithium hydroxide, its contribution is different from that of other strong bases. The saturation concentration of lithium hydroxide solution at normal atmospheric conditions is about 5.39 mol/L due to the solubility problem. It can be seen that the amount of activator added in the crystallization alkalinity interval of the lithium geopolymer is supersaturated, which leads to a constant pH in the geopolymeric reaction for a period of time. In addition, there will be water formation and evaporation in the reaction itself. Therefore, the amount of water added is only related to whether geopolymer form slurries. Figure 1b also proves that when the H_2_O/Li_2_O was increased by one, the diffraction peak intensity was significantly weakened. This oversaturation of the alkali can also explain the phenomenon of the intensity of the XRD diffraction peak being more or less consistent in several regions. In summary, the effect of these alkalinity levels on the transformation may be attenuated by the saturated nature of the activator, which is essentially the balance between the contribution of Li^+^ and the microcrystal induction effect and the influence of the gel amount on the crystallization.

Thus, the Li/Al ratio was fixed at 1.1, while the curing time remained at 24 h. Figure 1b shows the transformation of the lithium-based geopolymer at different temperatures. It can be seen that no molecular sieve is generated when curing below 30 °C. Then, with the temperature reaching 60 °C, the crystallinity of the sample gradually increases. When the temperature is higher than 60 °C, the intensity of the Li-ABW characteristic diffraction peak is basically unchanged, which can be judged as the best formation temperature for lithium zeolite. However, for each sample, the crystallization limit is subject to its original composition; that is, compared with the curing time and temperature, the transformation of the geopolymer in this experiment mainly depended on the Li/Al ratio of the raw materials. Table 1 lists the comparisons between the preparation process of the Li-ABW in this experiment and other experiments. Compared with the process proposed in this chapter, it can be seen that the in-situ conversion method of geological polymers has great advantages, and geological polymers are excellent precursors of zeolite.

### 2.2. SEM Analysis

**Figure 2:**
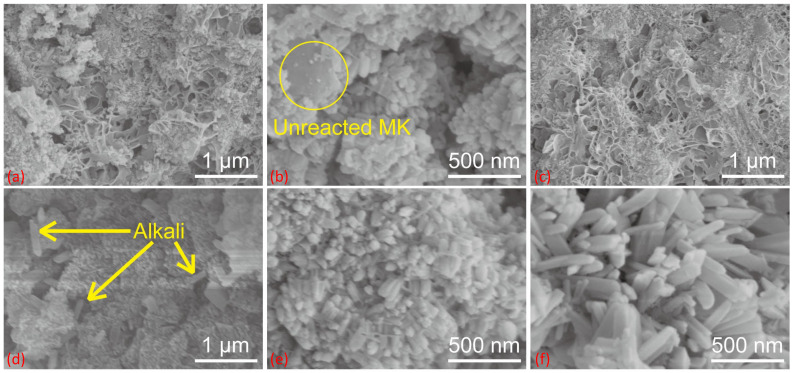
SEM patterns of (**a**) Li/Al = 1, T = 20 °C, (**b**) Li/Al = 1, T = 40 °C, (**c**) Li/Al = 0.6, T = 60 °C, (**d**) Li/Al = 1.7, T = 60 °C, (**e**) Li/Al = 0.9, T = 60 °C and (**f**) Li/Al = 1.1, T = 60 °C.

Figure 2 shows the SEM images of the geopolymer and Li-ABW zeolite. The fixed curing time is 24 h. Figure 2a shows Li/Al = 1.1, curing at 20 °C. The clear gel structure, incompletely reacted metakaolin participles, and a small amount of lithium zeolite microcrystals, which will become the nucleus of the subsequent gel transformation, can be seen. When the curing temperature rose, as shown in Figure 2b, with Li/Al = 0.9 and curing at 40 °C, due to the relatively insufficient amount of activator, large MK particles remained and zeolite crystals grew on its surface. Figure 2c shows the condition under Li/Al = 0.6 and T = 60 °C, at which the alkalinity is relatively insufficient. Compared with low-temperature curing, the amount of gel increases at high temperatures, and the unreacted raw materials basically disappear. However, there is no zeolite microcrystalline structure in the image. Accordingly, in Figure 2d, with Li/Al = 1.7 and T = 60 °C, the gel was relatively denser and the only crystal phase in the image may be an unreacted LiOH·H_2_O solid, which can also prove the influence of the alkalinity on the conversion of the geopolymer, as mentioned above.

In Figure 1, the XRD images with Li/Al = 0.9–1.1 are basically the same in terms of the intensity, but differ in microstructure. As shown in Figure 2e,f, it can be seen that the molecular sieve from the crystallization of the geopolymer was at the nanoscale, while the Li-ABW molecular sieve prepared through hydrothermal reaction was mostly at the micrometer scale [27,28,29,30,31,32]. It has been proven that, under the appropriate conditions, the geopolymerization reaction will produce microcrystals that are favorable for zeolite formation. Overlapped zeolite crystals can also be observed, which are common in reports of geopolymer conversion [32,33,34]. In terms of stacking, the microstructure of Li/Al = 0.9 with a relatively small amount of activator is relatively dense, and Li/Al = 1.1 is the opposite, but has the accumulation of a larger single crystal caused by the high alkalinity in the morphology. It is possible that more activators lead to an increase in the amount of gel, which are essential precursors for molecular sieves, and that the corresponding amount of Li^+^ content may lead to an improved induction of zeolite formation.

### 2.3. In-Situ Infrared Spectroscopy Analysis

**Figure 3:**
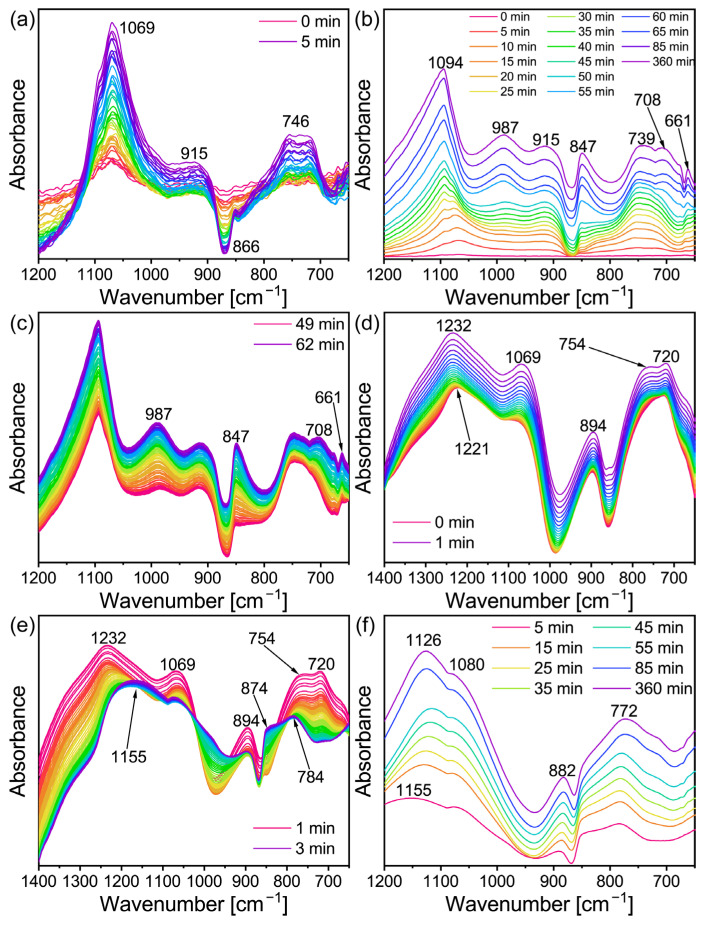
In situ DRIFTS of (**a**) Li/Al = 1.1, t = 0–5 min (**b**) Li/Al = 1.1, t = 0–360 min, (**c**) Li/Al = 1.1, t = 49–62 min, (**d**) Li/Al = 0.6, t = 0–1 min (**e**) Li/Al = 0.6, t = 1–3 min and (**f**) Li/Al = 0.6, t = 5–360 min.

In order to further explore the conversion mechanism of the lithium geopolymer, the slurry was analyzed using in-situ infrared spectroscopy. Figure 3b shows the in-situ infrared image of Li/Al = 1.1 and T = 60 °C when the reaction time is 0–360 min. Using the same recording method as in Figure 3b,f shows the image of Li/Al = 0.6. As the curve displacement of the equipment led to its absorption peak strength rising slowly with the passage of time (which can be seen from Figure 3c), the curve was made independent and was analyzed in the experiment. For the geopolymer, the fingerprint area of 800–1200 cm^−1^ is more significant. In addition, there are many vibration modes of Si-O-Si in the raw materials (including asymmetric stretching vibration, symmetric stretching vibration, and bending vibration) within 370–1300 cm^−1^ [35]. In conclusion, combined with the instrument parameters, the measuring range was 650–1200 cm^−1^. It can be seen from Figure 3a,b that the absorption peak first appeared at 1069 cm^−1^ in 0–5 min, shifted to 1078 cm^−1^ in 30 min, and then gradually disappeared and merged into the strong 1094 cm^−1^. The vanishment of 1069 cm^−1^ is considered to be the dissolution of the Si-O-Si bond in the MK under the action of alkali [36].

The aluminum in the Al-O bond in the MK is bonded with the six-coordination, while in the Al-O tetrahedron, it formed after alkali excitation in the four-coordination, and [AlO_4_] occupies the original [SiO4] after polycondensation, which leads to the change in the chemical environment of Si and Al [37]. For the Na-activated MK, the absorption peak of the stretching vibration of the Si-O-T (T = Al or Si) bond generally appears at 1076 cm^−1^ [37,38], however, it may shift due to the use of lithium hydroxide as the activator. In general, the peak at 1095 cm^−1^ corresponds to the asymmetric vibration of the Si-O bond. In addition, the absorption peak of the aluminum-rich phase Q3 at the initial reaction stage corresponds to 1093 cm^−1^ [35]. Therefore, the strong peak at 1094 cm^−1^ may be the result of the joint contribution of the above three factors. The absorption peak at 746 cm^−1^ may be the joint action of the stretching vibration of the Al-O bond [39] and the symmetric stretching vibration of Si-O-T, which generally occurs between 600–800 cm^−1^ [35]. The increase in the intensity of the absorption peak here confirmed the dissolution of the six-coordinate Al in the geopolymerization reaction. The peak that appeared at 915 cm^−1^ might correspond to the asymmetric expansion of the Si-O-T bond, which is active in the geopolymeric reaction [35]. The generation of zeolite led this toward the high wavenumber. However, the infrared spectrum changed with time.

**Figure 4:**
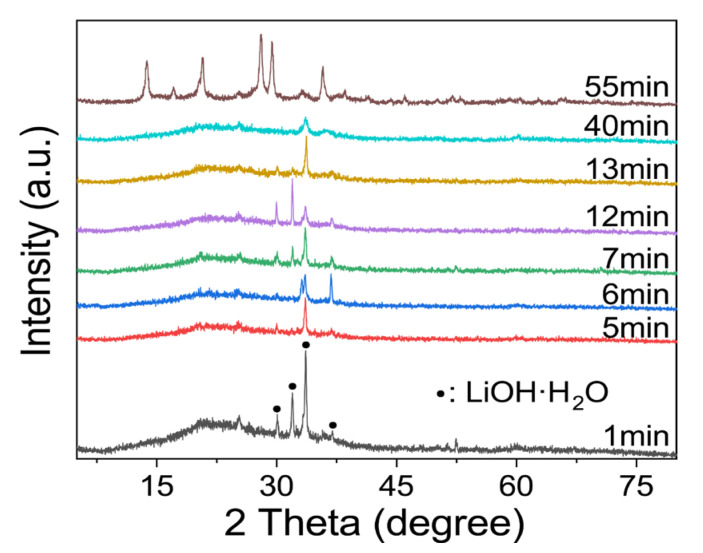
XRD characterization of Li/Al = 1.1 with different curing time.

There were obvious changes in the in-situ infrared image after approximately 50–60 min, as can be seen in Figure 3b. As shown in Figure 3c, the 49–62 min ATR-FT-IR image was separated. There are obvious adsorption peaks at 987 cm^−1^, 847 cm^−1^, 708 cm^−1^, and 661 cm^−1^, and there is no sharp change when the reaction lasts for 360 min (the top curve in Figure 3b). On one hand, the geopolymer slurry is actually solidified during the induction period of zeolite formation [40], which means that the transformation may occur during curing if the relevant conditions are suitable. On the other hand, these peaks appeared almost simultaneously. This indicated that the geopolymer may have been transformed at this time point. In addition, Figure 4 can also prove this inference, even if the intensity of the characteristic peak is low. At the beginning of the reaction, a relatively excessive amount of lithium hydroxide monohydrate (JPCDS card No. 01-075-0883) was detected in the sample, which also proved that the low-temperature cryogenic method of liquid nitrogen can suspend the geopolymerization reaction at the corresponding time point. As can be seen in the figure, with the progress of the geopolymerization reaction, the lithium hydroxide monohydrate continues to dissolve, its characteristic peak gradually disappears, and the entire system transforms into an amorphous geological polymer. The characteristic peak of the Li-ABW-type molecular sieve appears until the reaction proceeds to 55 min. It can be seen from this that, under the conditions of this experiment, when the reaction time is more than 55 min, the crystallization of the zeolite is actually carried out synchronously with the solidification and curing of the geopolymer. Moreover, the absorption peak at 746 cm^−1^ shifts towards the low wave number during the reaction; this could be interpreted as a change in the bond length and angle of the Si-O-Si in the gel [41].

In contrast to the transformation group, the change in the absorption peak in the geopolymer group was mainly concentrated in the 1–3 min period. In the case of the rising relative intensity displacement between the curves (Figure 3c), the absorption peak in Figure 3e shows strength weakening. This means that the corresponding raw material was involved in the reaction and was consumed. In addition, the peak shifted and weakened due to the reduction in other vibration contributions, and the relevant absorption peak may be a part of the original synthetic broad peak. There are two aspects that can support this point: Firstly, the shift from 1069 cm^−1^ to 1080 cm^−1^, which might be due to the joint contribution of the Al dissolution to increase the signal of the asymmetric stretching of the Al-O bond at 1085 cm^−1^ [35] and the consumption of MK in the reaction process. Lastly, two absorption peaks (720 cm^−1^ and 754 cm^−1^) corresponded to the disappearance of the MK. A new peak was separated at 784 cm^−1^, which may be the symmetric stretching vibration of Si-O-T [38].

Figure 3f shows that the absorption peak of the geopolymer sample with Li/Al = 0.6 changed little with time and only partially shifted, which also proved that the new peaks in Figure 3b represented the formation of a lithium molecular sieve. The absorption peak that finally shifted at 1126 cm^−1^ requires further discussion; it shifted from 1221 cm^−1^ at the beginning of the reaction to 1232 cm^−1^, then shifted to 1116 cm^−1^ as the reaction proceeded, and ultimately returned to 1126 cm^−1^. The reason for the first shift to the high wavenumber is the stretching vibration of the Al-O band [39]. The absorption peak at 1221 cm^−1^ may correspond to the stretching vibration of the Si-O-Si bond of Q0 [35] as the Si/Al of MK is close to one, Si and Al were dissolved at the same time in the initial reaction, and the gel was relatively rich in silicon; therefore, the main peak will shift to the high wavenumber [42]. The subsequent situation, from high to low then to high, might be due to the relatively faster dissolution of aluminum in the MK during the geopolymerization process, which generates more aluminosilicates, causing the main peak of Si-O-T to shift to a low wavenumber. As the reaction progressed, further silicon dissolution improves the degree of cross-linking, which causes the main peak to shift back [42]. This is further supported by the work of A. et al. [43], who used sodium hydroxide to activate F-type fly ash and proved that Al-rich gel was first generated at the initial stage of the activation. The silicon source was gradually dissolved and the gel was transformed into a silicon-rich phase with the extension of the curing time.

**Figure 5:**
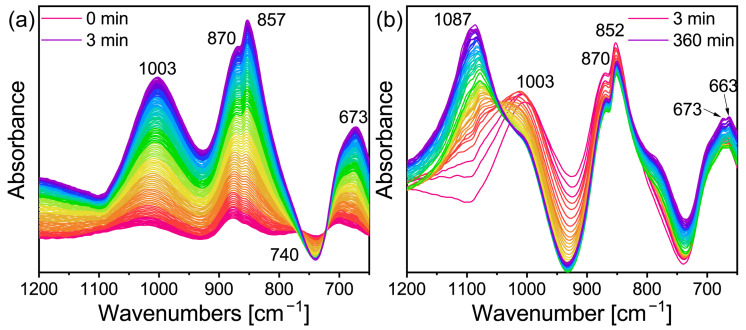
In situ DRIFTS of (**a**) Li/Al = 1.7, t = 0–3 min (**b**) Li/Al = 1.1, t = 3–360 min.

The in-situ infrared image of the lithium-based geopolymer under high alkali conditions (Li/Al = 1.7) is shown in Figure 5a,b. It can be seen that in the case of an excessive addition of lithium hydroxide monohydrate, the image at the initial stage of the geopolymerization reaction is significantly different from those in Figure 3a,d. Firstly, the absorption peak at 1040 cm^−1^ may be caused by the Si-O-Si stretching vibration of unreacted metakaolin [44,45,46]. It then shifts to a low wavenumber, of 1003 cm^−1^, which may be related to the superposition of the asymmetric stretching peaks representing the Si-O-T (T = Si, Al) bond at 986 cm^−1^ and 993 cm^−1^, as well as the asymmetric stretching peaks [47] of the Si-O-T (T = Si, Al) bond of Q2 generated at the initial stage of the reaction. Secondly, the absorption peak appears at 870 cm^−1^ may represent the end vibration of Si-O [48], which is reflected in both Li/Al = 0.6 and 1.1, which is also an example of the absence of zeolite formation. In addition to the absorption peak mentioned above, the absorption peak at 857 cm^−1^, which occurs later than 870 cm^−1^, may be related to the asymmetric stretching vibration of the Si-O bond. Finally, the absorption peak appearing at 673 cm^−1^ may be related to the Si-O-T (T = Si, Al) [49] and the vibration of the O-Si-O bond. The decrease in the intensity at 740 cm^−1^ may be caused by the complete dissolution of metakaolin in the presence of excessive sodium lithium hydroxide addition.

As the reaction continues, as shown in Figure 5b, the absorption peaks at 870 cm^−1^, 852 cm^−1^, and 673 cm^−1^ that appear in Figure 5a do not change significantly. The changes in the image are as follows: First, the main peak at 1003 cm^−1^ shifted and the intensity decreased, and finally it shifted to 1087 cm^−1^ at the end of the reaction. Similarly to the cause of the shift of the main peak in Figure 3b, when the amount of lithium hydroxide is sufficient, the absorption peak corresponding to the asymmetric stretching vibration of the Si-O-T bond at 1076 cm^−1^ [48], the asymmetric contraction of the Al-O bond at 1085 cm^−1^ with the dissolution of aluminum, the asymmetric stretching vibration of the Si-O bond at 1095 cm^−1^, the absorption peak of the aluminum-rich phase Q3 appearing at 1093 cm^−1^ [35], and the chemical environment of Si and Al change [37]. Factors such as the vibration of Si-O [50] and the presence of Q3 bonds [51] jointly lead to the occurrence of an absorption peak at 1087 cm^−1^. The difference is that, due to the absence of zeolite formation, there is no blocking effect on the surface of the geopolymer, resulting in the detection of the signal of the asymmetric stretching peak [47] of the Si-O-T of Q2 in the geopolymer; therefore, the absorption peak at 1003 cm^−1^ remains in the image. In addition, the occurrence time of the absorption peak at 663 cm^−1^ is similar to that at 661 cm^−1^ in Li/Al = 1.1, which may be related to the crystal plane corresponding to the characteristic peak of 35.81° in the XRD image in Figure 1a.

In summary, the following conclusion has been proposed: On one hand, the conversion time of lithium-based geopolymers is about 50 min from the beginning of the geopolymerization, which to some extent supports the theory that the conversion time of in-situ zeolite may be earlier than the solidification time of the geopolymer. On the other hand, the low alkali system shows the consistency of the in-situ infrared curve, and the complexity of the changes are most active at 1–3 min. In contrast, the transformation group and the high-alkali system show good inducibility at the initial stage of the reaction, which may be related to the inductive effect of zeolite microcrystals on the gel of the geopolymers.

### 2.4. XPS Analysis

**Figure 6:**
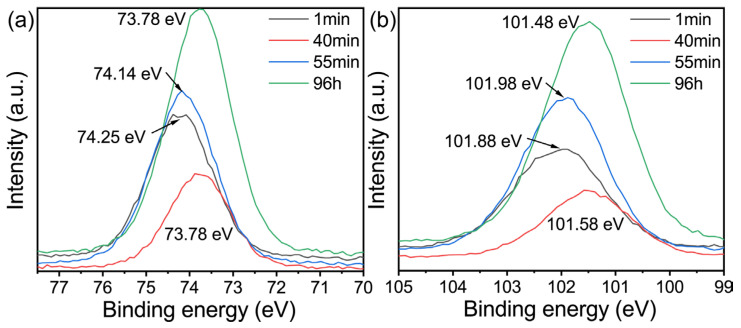
High resolution XPS spectra of (**a**) Al (2p), (**b**) Si (2p).

**Figure 7:**
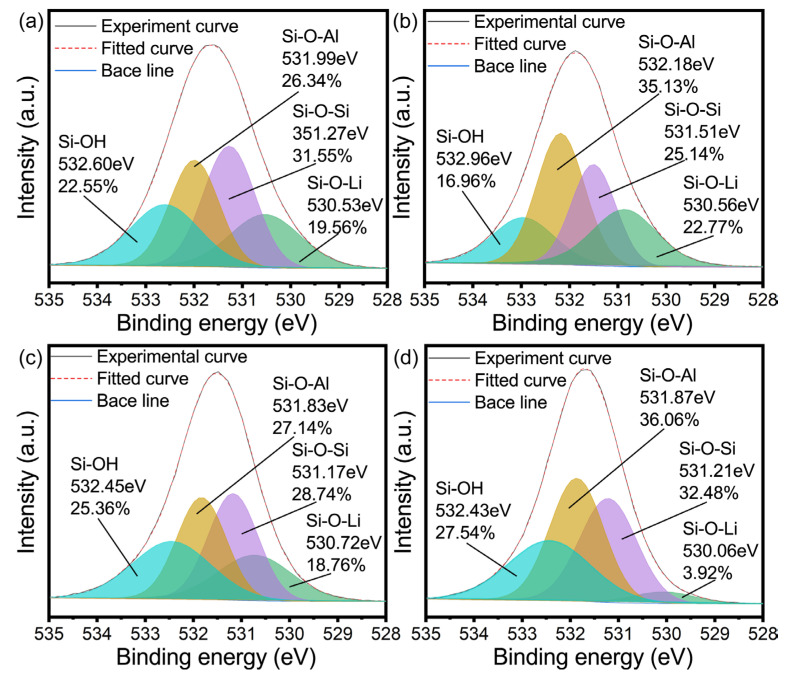
O 1s high resolution XPS spectra of (**a**) 1 min, (**b**) 40 min, (**c**) 55 min and (**d**) 96 h.

To further determine the formation mechanism of lithium zeolite, as shown in Figure 6 and Figure 7, the elemental composition of the initial sample (reaction time: 1 min), the pre-conversion sample (40 min), the initial conversion sample (55 min), and the completely converted sample (reaction time: 96 h) were detected using XPS.

According to a previous report [27,52], there are usually four chemical forms of samples in geological polymers and zeolites. Therefore, when performing peak fitting on the O 1s high-resolution spectrum, Si-OH, Si-O-Al, Si-O-Si, and Si-O-Li are selected. From the beginning of the reaction until the conversion of the geopolymer, the electron binding energy decreased, with Al decreasing from 74.25 eV to 73.78 eV, while Si decreased from 101.88 eV to 101.58 eV, which may be due to the increase in the release of Si and Al elements from the metakaolin, indicating that geopolymerization is mainly carried out.

With the increase in the curing time, the electronic binding energy of Al and Si increases at the initial stage of zeolite conversion, which may be because the lithium-based geopolymer reaction system needs to go through a process from disorder to order during crystallization, one of which involves the lithium entering into the geopolymer gel structure, which will enter into the zeolite skeleton in the form of a lithium ion during the process of geopolymer crystallization into zeolite. Combining this with Figure 7c,d, it can be seen that the content of Li-O-Si decreases sharply during the transformation of the lithium-based geopolymer. On the other hand, the geopolymer gel should rearrange the microcrystals generated in the geopolymerization as the crystal nucleus to produce Si/Al close to one and to generate zeolite. Both of the above may contribute to the increase in the electron binding energy of Al and Si. At the same time, the increase in the electron binding energy before and after transformation also reflects the fact that the change of the geopolymer gel system is different from the formation of geopolymer gel, which to some extent proves the formation of the boiling stone phase in this process. With the increase in the crystallization degree, the total energy of the system decreases. At this time, Al and Si exist in the form of a zeolite skeleton. In this case, compared with the geopolymer gel, its chemical environment is more stable. Therefore, the electronic binding energy of Al and Si decreases continuously during the reaction process and is ultimately less than the electronic binding energy of Al and Si in the initial reaction of the geopolymer.

From the peak fitting of the high-resolution spectrum of O 1s, as shown in Figure 7, the following inference can be drawn: Firstly, it can be seen that the content of Si-O-Al significantly increased from the beginning of the reaction to the beginning of crystallization, which may be due to the relatively faster dissolution rate of Al in the early stage of the geopolymerization reaction. Secondly, Figure 7b,c show that the changes in the content of the Si-O-Al and Si-O-Si bonds before and after the formation of zeolite. With the dissolution of more Si, and under the control of the main crystal form Li_4_Al_4_Si_4_O_16_·4H_2_O (this can be seen from XRD images), the content of these two bonds tends to be consistent. Finally, it can be seen from Figure 7d that after deducting the content of Si-O-Li corresponding to Si-O-Al in the gel, the content of the Si-O-Si and Si-O-Al bonds is almost the same, which also explains the contribution of the zeolite transformation in Figure 7c to the convergence of the content of the Si-O-Si and Si-O-Al bonds.

Geopolymerization generally reduces the energy of the system, while the crystallization process itself increases the energy of the system. However, after complete crystallization, the overall energy is lower than that of the system in the state of the geopolymer, which to some extent proves that the geopolymer is a good precursor for zeolite conversion. The crystallization process of the geopolymer-based zeolite is accompanied by the process of Li separating from the L-A-S-H gel and entering the zeolite skeleton. In other words, in the process of geopolymer conversion, the rearrangement of the Si and Al gel and the separation of Li belong to two steps, respectively, and are not directly converted with L-A-S-H as the raw material. 

### 2.5. TG-DTG Analysis

**Figure 8:**
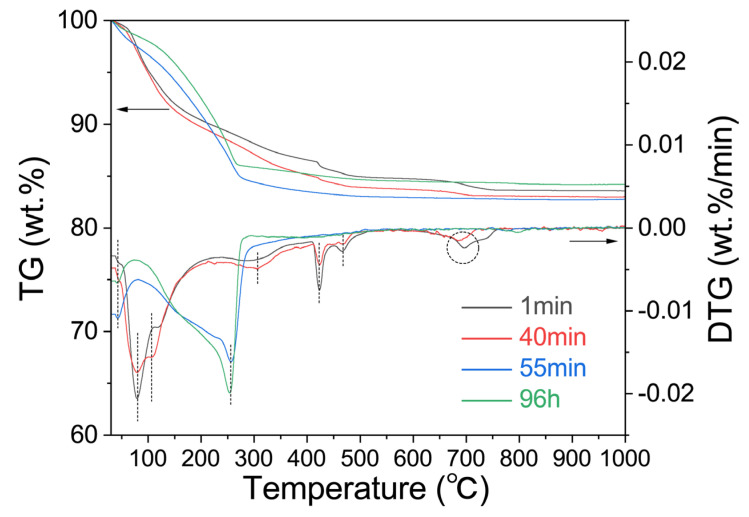
TG-DTG characterization of Li/Al = 1.1 with different curing time.

Figure 8 shows the TG-DTG curves of the samples with reaction times of 1 min, 40 min, 55 min, and 96 h. According to the figure, there are three weight loss intervals for the geological polymer samples: 25–200 °C, 200–500 °C, and 600–800 °C, which correspond to the mass loss peak of the geopolymer gel L-A-S-H, hydrotalcite [53], a mass loss peak in which the lithium hydroxide monohydrate loses crystalline water and decomposes, and a mass loss peak in lithium carbonate, respectively. The weight loss range of the molecular sieve samples mainly occurs at 100–300 °C, and the corresponding crystal form of the molecular sieve collapses from Li_4_Al_4_Si_4_O_16_·4H_2_O to Li_2_Al_2_Si_2_O_8_·0.18H_2_O after water loss. It can be seen that the formation of zeolite has significantly changed the properties of the lithium-based geopolymer: First, in general, the zeolite generated in situ by the geopolymer usually grows on the surface of the geopolymer gel, and the zeolite generated in situ completely seals the L-A-S-H gel to keep it unchanged in the weight loss temperature range of the unconverted lithium-based geopolymer. Secondly, the formation of zeolite induced the formation of geopolymer gel to a certain extent, making the original weight loss segment of the hydrotalcite disappear. Finally, the addition of lithium hydroxide monohydrate is relatively excessive, while the conversion group has no significant changes in the corresponding dehydration and decomposition stages of the lithium hydroxide monohydrate, which may also be related to the fixation of the zeolite on the alkali.

The formation of geopolymer-based lithium zeolite will have an impact on the geopolymer system. The transformation of the zeolite on the surface of the geopolymer will seal the L-A-S-H gel, as well as the excess alkali in the gel pore solution or system, which will fix the excess alkali later.

## 3. Conclusions

In this paper, Li-ABW zeolite was prepared in situ under non-hydrothermal conditions. The results showed that the optimum Si/Al ratio formed by a molecular sieve is 1.1. The geopolymer can be transformed at 30 °C, and the optimal temperature is about 60 °C. The formation of zeolite was characterized using in-situ infrared spectroscopy. The in-situ crystallization took place approximately 50 min after the reaction under the above optimal conditions. This work characterized the induction of zeolite microcrystals on a geopolymer gel, characterized the process of the in-situ transformation of zeolite using a geopolymer, and proved that geopolymer can be used as a good precursor of zeolite. In summary, this experiment provides a simple process for the preparation of lithium zeolite with lower energy consumption, which might be applied as a solid electrolyte.

## 4. Materials and Methods

### 4.1. Materials

SiO_2_ (51.02 wt.%), Al_2_O_3_ (44.99 wt.%), etc. (3.99 wt.%) in metakaolin were provided by Inner Mongolia Super New Material Co., Ltd Inner Mongolia, Hohhot, China. Lithium hydroxide monohydrate (AR) was purchased from Shanghai Aladdin Biochemical Technology Co. (Shanghai, China) Deionized water was provided by the School of Chemistry and Chemical Engineering, Guangxi University.

### 4.2. Characterization of the Samples

The synthesis steps of the Li/Al = 1 geopolymer-based lithium molecular sieve are as follows: 100 g metakaolin was mixed with 37.68 g LiOH·H_2_O and added to a certain amount of deionized water (due to the low solubility of LiOH, the study of H_2_O/Li_2_O lost its significance. In this experiment, the slurry capable of forming a geopolymer was used as the adding standard of water quantity, which was approximately consistent with the quality of metakaolin used), then the mixture was stirred evenly with dispersant. The slurry was cast into a disk mold and cured at 60 °C for 24 h. After demolding, the geopolymer-based Li-ABW was obtained. Other samples were prepared in the same manner as described above, changing the curing temperature, time, and Li/Al ratio. Most samples used for testing are freeze-dried, the samples at the corresponding time point (accurate to the minute) underwent cryogenic treatment with liquid nitrogen and were then freeze-dried.

### 4.3. Synthesis of the Zeolites

The X-ray diffraction (XRD) characterization of the samples was performed with Rigaku MiniFlex 600 (Tokyo, Japan) using Ni filters and Cu (Kα) radiation (λ = 1.392 Å) at a voltage of 40 kV and current of 15 mA. The scanning range(2θ) was between 5 and 80°, under the conditions of 0.02° step size, 0.5 s dwell time, and 5°/min scanning rate. The morphology of the geopolymer gel and zeolite was photographed using a field emission scanning electron microscope (FE-SEM) (Hitachi, SU8220, Tokyo, Japan) after gold sputtering. In-situ diffuse reflectance infrared Fourier transform spectroscopy (DRIFTS) in the range of 650~4000 cm^−1^ of the samples in an in-situ cell was recorded with a spectrometer (Thermo Fisher Scientific (Waltham, MA, USA) IS50. X-ray photoelectron spectrometer (XPS) characterization of the samples was performed with Thermo Scientific Nexsa. TG/TGA characterization of the samples was performed with a TGA2 thermogravimetric analyzer.

## Figures and Tables

**Table 1:** Comparison of the preparation process of Li-ABW with other experiments.

Starting Mixture	Temperature (°C)	Total Time (h)	References
MK-LiOH·H_2_O-H_2_O	60	24	This work
CFBFA-LiOH·H_2_O-H_2_O	180	24	[27]
FA-LiOH·H_2_O-H_2_O	160	16	[28]
FA-LiOH-H_2_O	180	24	[30]
FACs-LiOH·H_2_O-H_2_O	180	24	[31]

## Data Availability

Not applicable.
